# Implementing fencing as adapted physical activity in non-metastatic breast cancer patients: design and early rehabilitation strategy of the FENICE study protocol

**DOI:** 10.3389/fonc.2024.1407919

**Published:** 2024-08-09

**Authors:** Massimiliano Berretta, Daniele Garozzo, Calogero Foti, Mario Roselli, Marco Materazzo, Giulia Vita, Ferdinando Iellamo, Marco Scordari, Giordana Di Mauro, Giovanna Spatari, Alessandro Ottaiano, Annalisa Noce, Marco Pellicciaro, Alessia Bignucolo, Gianluca Vanni, Oreste Claudio Buonomo

**Affiliations:** ^1^ Department of Clinical and Experimental Medicine, University of Messina, Messina, Italy; ^2^ Department of Clinical Science and Translational Medicine and School of Sports Medicine, University Tor Vergata, Rome, Italy; ^3^ Physical and Rehabilitation Medicine, Tor Vergata University, Rome, Italy; ^4^ Medical Oncology Unit, Department of Systems Medicine, Tor Vergata University Hospital, Rome, Italy; ^5^ Breast Unit, Department of Surgical Science, PTV Policlinico Tor Vergata University, Rome, Italy; ^6^ School of Specialization in Medical Oncology, University of Messina, Messina, Italy; ^7^ Department of Biomedical and Dentistry Sciences and Morphological and Functional Imaging, University of Messina, Messina, Italy; ^8^ Istituto Nazionale Tumori di Napoli, Istituto di Ricovero e Cura a Carattere Scientifico (IRCCS) “G. Pascale”, Napoli, Italy; ^9^ Department of Systems Medicine, University of Rome Tor Vergata, Rome, Italy; ^10^ UOSD Nephrology and Dialysis, Policlinico Tor Vergata, Rome, Italy; ^11^ Department of Health Science, University of Basilicata, Potenza, Italy

**Keywords:** BC, surgery, rehabilitation, fencing, APA, multidisciplinary network

## Abstract

**Background:**

Improving prognosis of BC patients has drawn the attention of health care professionals on disease related long-term side effects and on the multiple treatments BC patients must undergo. Despite advances in procedures, surgery still has multiple detrimental effects, including pain, edema, and limited mobility. For this reason, fostering adapted physical activity (APA) and healthy lifestyle (including a balanced diet and weight management) should become an everyday purpose of healthcare professionals. Fencing may be a well-suited activity to counteract fatigue, pain, and limited arm mobility.

**Method and analysis:**

The FENICE study is a mono-center, randomized clinical trial targeting women with BC stages I-III within four weeks from BC surgery. Participants in the control arm will receive the usual recommendations based on the good clinical practice guidelines. In the study arm, participants will be treated with the usual clinical and therapeutic recommendations together with APA and correct lifestyle suggestions.

**Objective:**

The primary objective of the study is to compare whether implementation of APA and healthy lifestyle in BC patient after surgery will result in an overall improvement of physical and mental status.

**Conclusion:**

Fencing and its early application in postoperative period may represent a feasible strategy to be implemented in the rehabilitation journey of BC patients.

**Ethics and dissemination:**

The study protocol FENICE has been approved by an Italian Ethics Committee on May 2023 (R.S 100.23 5^th^ May 2023).

## Introduction

Breast cancer (BC) is a growing global public health concern. Over time, different risk factors have been identified: alongside the genetic mutations (most commonly involving BRCA1 and BRCA2) implicated in hereditary form of breast and ovarian cancer, the most important risk factors for the sporadic form include personal and family history of BC, wide fertile window (related to early menarche, older age at menopause, first pregnancy after the age of 30 and nulliparity), the exposure to external sources of hormones (during hormone replacement therapy or using oral contraceptives), dense breast tissue and lifestyle based risk factors as alcohol consumption, tobacco, physical inactivity and sedentary lifestyle (1–4). The cancer screening programs in Italy comply with the guidelines established at European level, which are contained in the EU recommendation of December 16, 2003. These guidelines identify the three screening programs (breast, uterine and colorectal cancer) as the most effective cancer prevention measures (5). Despite the national cancer screening program launched since 2005, BC still affects many women in Italy. According to the latest AIRTUM (Associazione Italiana Registri Tumori) report, almost 56,000 new cases of BC have been diagnosed in our country in 2023, representing 30% of female tumors and 13% of all tumors regardless of gender (6, 7). At the same time the survival rate of patients has significantly improved (88% of 5-year survival rate) due to the recent treatments progress and the number of cancer survivors raised from approximately 2 million in 2006 to 3.6 million in 2020 (AIRTUM – Associazione Italiana Registri Tumori) (7). Improving prognosis due to early diagnosis and advances in therapeutic treatments has drawn the attention of health care professionals on disease related long-term side effects and on the multiple treatments BC patients must undergo (8). However, surgery represents the standard treatment for loco-regional BC, while radiotherapy (RT), antiblastic chemotherapy (AC), target therapy, immunotherapy and endocrine treatment are used in adjuvant and metastatic BC settings. During the past decades, conservative surgery (quadrantectomy) has become the first choice whenever possible, limiting mastectomy in selected cases only. Despite advances in procedures, surgery still has multiple detrimental effects, including pain, edema, and limited mobility (9–11). Posture very often appears to be compromised after mastectomy because the surgical scar limits arm mobility (e.g., internal rotation and shoulder droop are common, typically causing back pain) (12). In addition, the removal of lymph nodes (both the sentinel procedure and axillary lymph node dissection) is cited as a common cause of limited mobility and postoperative pain. Moreover, cancer treatments are also responsible of numerous long-term side effects as specified in **Table 1** (8, 13–18). Particularly, the use of aromatase inhibitors has been associated with possible risk of arthralgia and bone health worsening (19, 20). These aspects negatively affect the health-related Quality of Life (QoL) of BC patients (21, 22). BC patients also reported low levels of Work Ability (WA) and reduced outcomes in terms of employments status, employability, and fitness for work (23, 24). Some randomized clinical trials (25, 26) have demonstrated the benefit of regular and appropriate Adapted Physical Activity (APA) during and after BC treatment, particularly in terms of improved health status, strength, QoL and physical function, and fatigue reduction (**Table 2**) (35, 36). Moreover, two meta-analysis highlighted an inverse relationship between post-diagnosis APA and cancer-related mortality in patients with breast cancer compared to patients practicing low or no physical activity (37, 38). From another study emerged that patients with ER/PR negative cancer who had been physically active before the diagnosis had a significantly reduced risk for breast cancer recurrence compared to physically inactive patients (39). In early BC and in metastatic BC setting has been recently demonstrated that physical activity integration is able to improve general and physical well-being and mental health (40). Accordingly, these non-pharmacological treatments that alleviate long term side effects should be rated as a public health priority, as their role is crucial in improving overall survival (OS) and QoL in BC, cancer survivors included. For this reason, fostering APA and healthy lifestyle (including a balanced Mediterranean diet and weight management) should become an everyday purpose of healthcare professionals (41). In this context, APA as fencing has proven its affinity and efficacy as a supportive activity both during and after the treatment of BC patients (30, 35, 42, 43).

**Table 1:** Side effects related to cancer treatments in breast cancer patients.

Late Side effects	Type of treatments				
	Chemotherapy	Endocrine therapy	Radiotherapy	Surgery	Target Therapy
**Arthralgia**	✗	✓✓✓	✗	✗	✗
**Bone health**	✓	✓✓✓	✓	✗	✗
**Cardiovascular disease**	✓✓	✗	✓	✗	✓✓✓
**Depression and anxiety**	✓✓✓	✓	✓✓	✓	✓✓
**Fatigue**	✓✓✓	✓	✓✓	✗	✓✓
**Lymphedema**	✗	✗	✓	✓✓	✗
**Metabolic disturbances**	✓	✓✓	✗	✗	✓
**Neurocognitive dysfunction**	✓	✗	✓	✗	✓
**Pain**	✓	✗	✓✓	✓✓✓	✗
**Peripheral neuropathy**	✓✓✓	✗	✗	✓	✗
**Respiratory impairment**	✓	✗	✓✓	✗	✓
**Sexual disfunction**	✓	✓✓	✗	✗	✗
**Shoulder limited mobility**	✗	✗	✓	✓✓	✗

✓✓✓Probably related (data from RCTs); ✓✓Might be related (data from RCTs with smaller samples); ✓Could be related (single-arm studies); ✗No sufficient data.

**Table 2:** Beneficial effects of Adapted Physical Activity in cancer patients.

Sport/physical activity	Benefit	Reference
**Aerobic training**	**↓**CRF, **↑**health related QoL and physical function, **↓**anxiety, depression, **↑**sleep quality.	(27)
**Cycling**	**↑**QoL, **↑**Physical functioning, **↑**general health, **↑**vitality.	(28)
**Dragon Boat**	↑QoL, ↑Physical functioning, ↓fatigue.	(29)
**Fencing**	**↑**immunity function, **↑**adherence to therapies, **↑**health related QoL, **↓**CRF, **↓**anxiety, **↑**of the functional capacities.	(30)
**Resistance training**	**↓**CRF levels, **↑**health related QoL and physical function, **↓**lymphedema, **↑**aspects related to bone health.	(31)
**Rowing**	**↑**upper extremity and hip range of motion, **↑**lower and upper extremity strength, **↑**aerobic capacity and heart rate at rest and after prolonged effort.	(32)
**Swimming**	**↑**Flexion and external rotation of the operated arm.	(33)
**Walking**	**↓**Of the BMI, **↓**breast symptoms and in the arms, **↑**MS at the extremity of the upper limbs, **↓**of pain.	(31)
**Yoga**	**↑**joint mobility, **↑**muscle strength, **↑**QoL.	(34)

↓reduce; ↑ improve; CRF, cancer related fatigue; QoL, quality of life; BMI, Body mass index; MS, Muscle Strength.

Fencing can be a good activity to reduce fatigue, pain and limited arm mobility. As it is also a sport that naturally strengthens willpower, practicing fencing can have a positive effect on the attitude and psychological state of participants. Hence, the FENICE study protocol here described will assesses both physical and psychological outcomes.

## Research hypothesis and objectives

### Hypothesis

**Figure 1:**
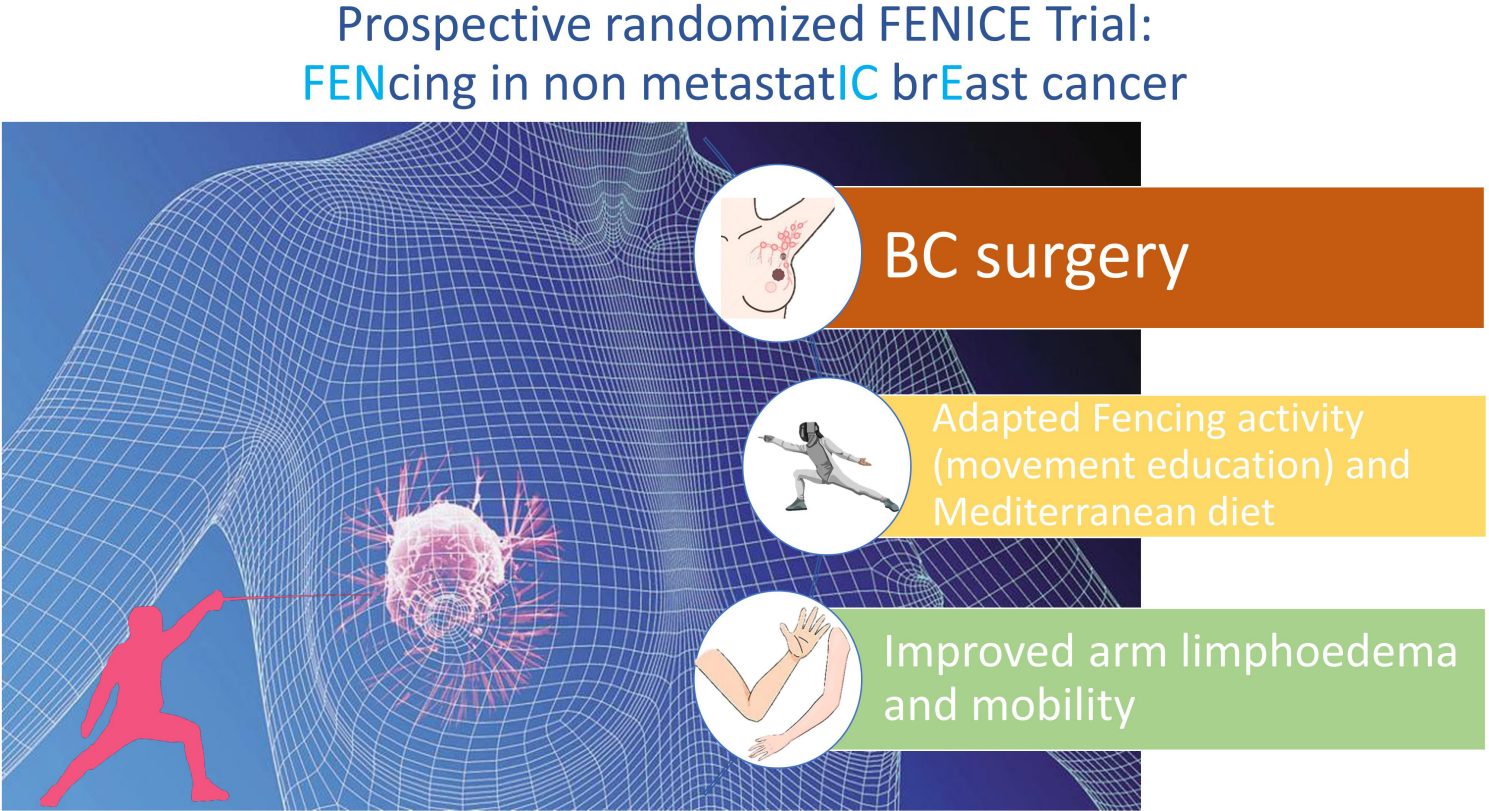
Graphical summary of the primary aim of FENICE trial.

Adapted physical activity along with the introduction of Mediterranean diet and their early application in postoperative period may be a successful strategy to improve the physical and psychological health of BC patients underwent surgery (**Figure 1**).

### Primary objective

The primary objective of the study is to compare whether implementation of an APA and healthy lifestyle (Mediterranean diet) will result in an overall improvement of physical and mental status including reduction in the number and severity of clinically relevant adverse events (AEs), in the study group compared with the control group during a 24-month enrollment period.

### Secondary objective

The secondary aim is to compare whether APA improves perception of cancer-related fatigue (CRF) and sleep quality (both measured via specific questionnaires), BMI, bone health, aerobic capacity, heart rate and serum lipid profile in the study arm. In addition, levels of Work Ability as well as health care provider and patient attitudes toward APA as adjunctive therapy will be assessed. Finally, the effect of adapted physical activity and a healthy diet on reducing the recurrence of the disease is being investigated.

## Methods and analysis

### Adaptation of fencing activity

Considering the high technical component of foil and saber, two fencing specialties, an adapted form of fencing is proposed that allows participants to enjoy the training more. As it is a one-sided sport, participants are encouraged to change the grip of the weapon several times during training in order to use both limbs equally and not to neglect the operated side. To avoid possible complications, low weight foil and saber are used within the APA. The wide use of parries forces the fencer to make larger movements that allow greater involvement of the shoulder joint to improve the range of movement of the operated arm. The content was developed by the Università degli Studi di Roma Tor Vergata in collaboration with Daniele Garozzo, specialist in sports medicine and Olympic foil champion; Fabio Maria Galli and Lucio Landi, foil, and saber coaches of Olympic fencers, respectively. From an athletic performance perspective, it must be considered a moderate-intensity physical activity (i.e., participants feel increased heart rate, respiration, and body temperature, but are still able to carry on a conversation), with the added benefit of being safe, psychologically engaging, fun, and social to encourage broad participation and minimal dropout (44).

### Design of FENICE trial

**Figure 2:**
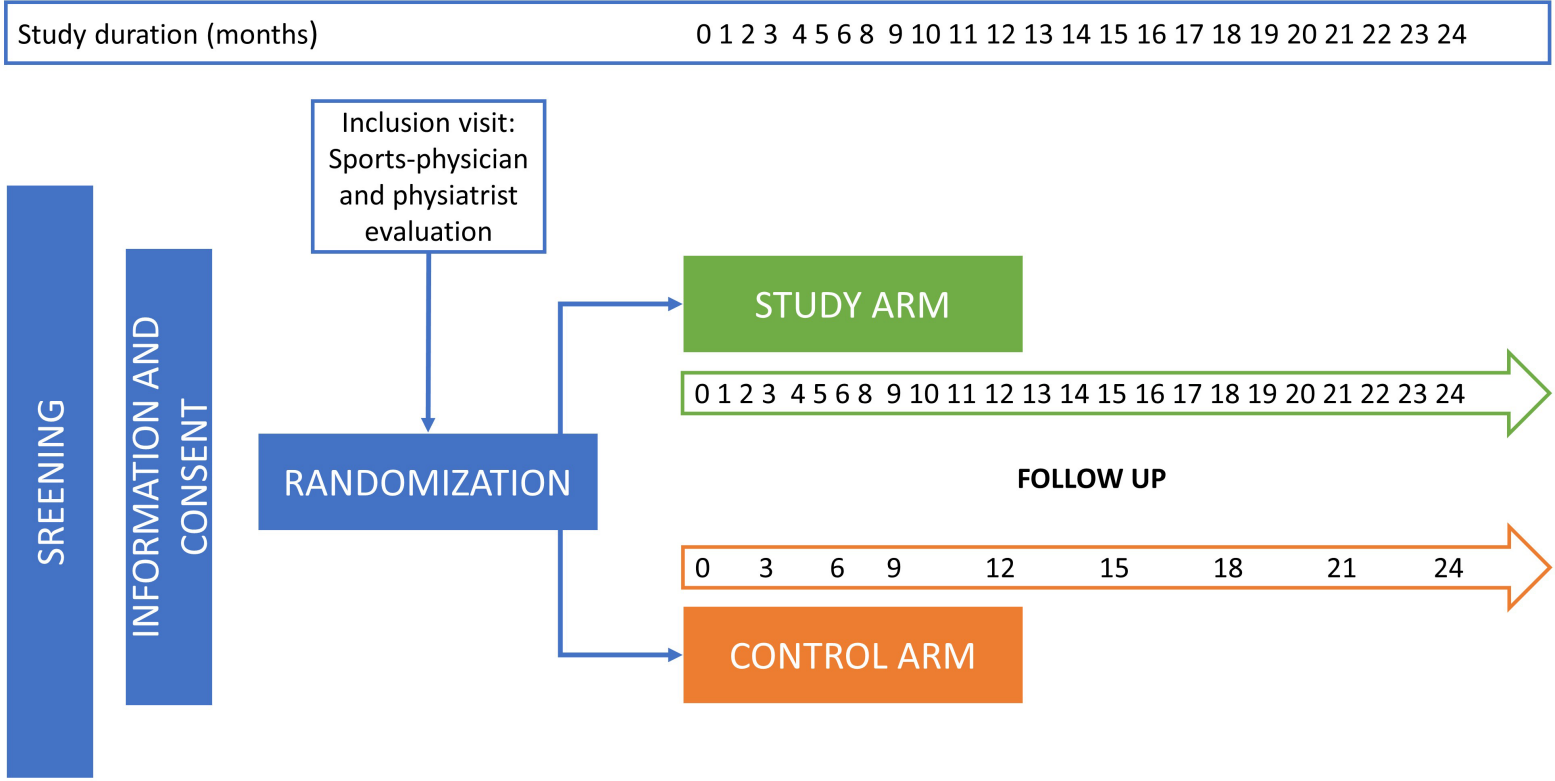
Study design of the mono center randomized FENICE clinical trial. All screened patients will receive a complete clinical, psychological, and instrumental evaluation, (lifestyle, comorbidities, oncological treatments and their related sequelae and AEs). Participants in the control arm will receive the usual recommendations based on the good clinical practice guidelines and according to 1) the stage of BC disease; 2) planned oncological treatments; 3) sequelae and AEs caused by oncological treatments. In the study arm, participants will be treated with the usual clinical and therapeutic recommendations together with APA and correct lifestyle suggestions (Mediterranean diet). All participants will be followed up for a minimum of 12 month, and up to a maximum of 24 months.

The FENICE study is a mono-center, randomized clinical trial targeting women with stages I-III BC within four weeks from surgery, conducted at the Breast Unit of Policlinico Tor Vergata Rome and Frascati Scherma ASD. The sequence of study and control group is randomized to minimize the influence of time-dependent variables. The participants, regardless of arm dominance, will be followed up by oncologists, radiotherapists, and surgeons according to the BC guidelines. All subjects participating in the study will undergo a sports medicine examination to objectify whether there are any alterations or diseases that pose a risk to the patient and to determine whether the patient needs to have additional tests and more specific examinations performed. For participants in the study arm only, APA consists of two fencing sessions (1 h/session) per week within 15 days of enrollment. The minimum number of sessions for the assessment of the primary endpoint is 48 sessions over six months up to a maximum of 96 sessions over 12 months (**Figure 2**).

After a warm-up period dedicated to muscle awakening and progressive cardio-respiratory stress, participants begin specific fencing exercises, starting with basic footwork (advance, retreat, lunge) and ending with actions in which the injured side holds the saber. During the main activity (40 minutes of fencing), exercise intensity will be individually tailored based on a preliminary assessment. All participants will undergo an exercise stress test on a cycle ergometer during the recruitment phase of the study. This test will measure key physiological parameters, including heart rate, oxygen consumption (V’O2), and the MET (Metabolic Equivalent of Task) values achieved (45). The MET value, which represents the energy cost of physical activities, will be used to calibrate the intensity of physical exertion during training sessions. Specifically, the fencing exercises will be designed to reach an intensity that is approximately 60-70% of the participants’ maximal MET values determined during the stress test. This range corresponds to moderate-intensity exercise, which has been shown to be effective in improving cardiovascular fitness and overall health. Additionally, to ensure safety and maximize adherence, participants’ heart rates will be monitored during training to maintain the desired intensity. The target heart rate zone will be calculated using the Karvonen formula, which considers the participants’ resting heart rates and the intensity percentage derived from their stress test results. This individualized approach ensures that participants engage at a cardio-respiratory intensity that is challenging yet enjoyable. They will be encouraged to maintain a level of exertion where they can still respond verbally (the “talk test”), indicating they are working within a safe and effective moderate-intensity zone (46–48).

**Figure 3:**
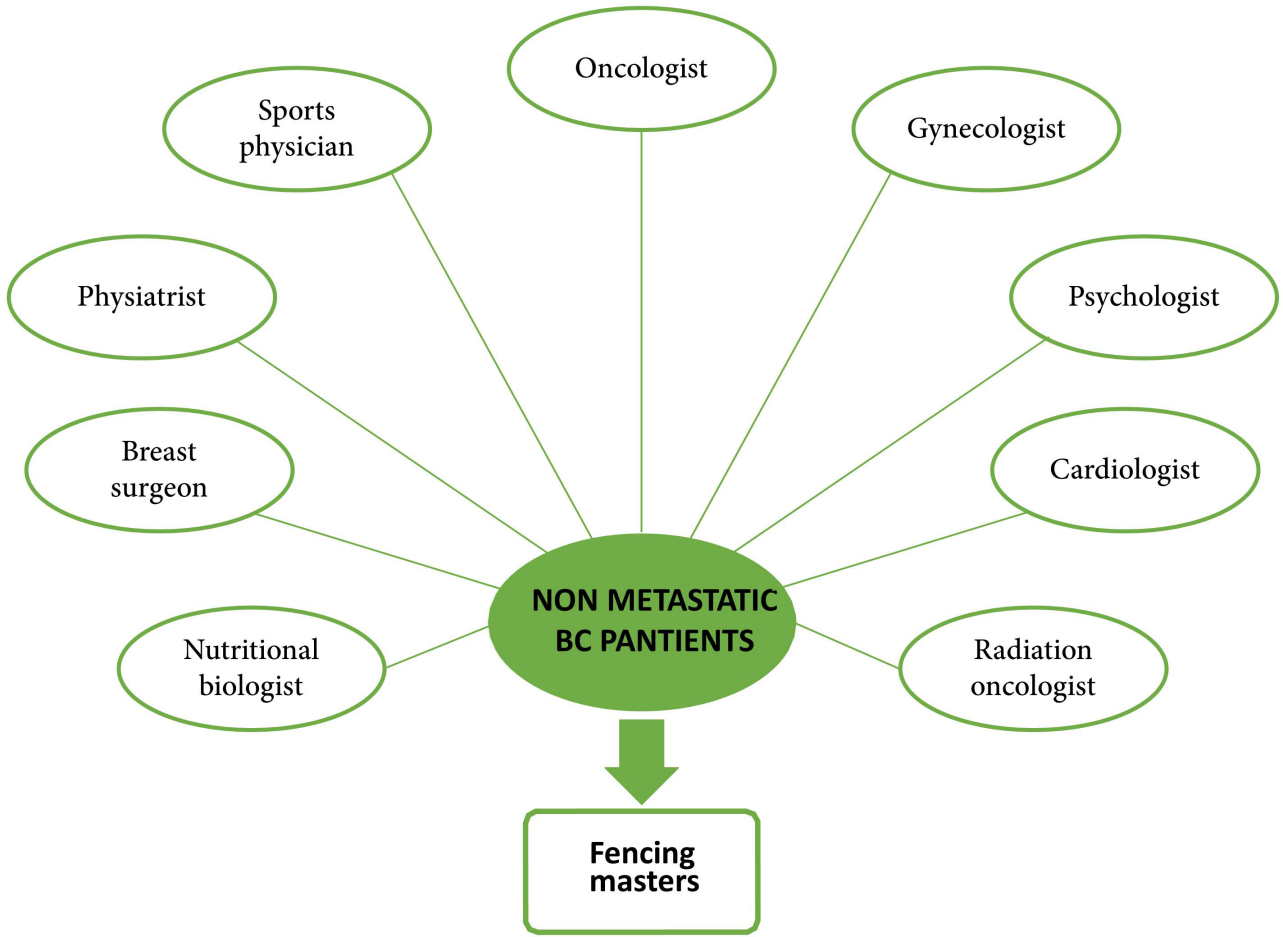
Specialists composing the multidisciplinary team of the FENICE clinical trial. Each BC patient enrolled in the study arm will be evaluated and monthly followed up by specialists before and during the adapted fencing activity.

Adapted fencing includes offensive and defensive actions, which are performed considering the mobility of the shoulder. The primary (tierce = outside; quart = inside; fifth = head) and secondary blocks are therefore performed by patients with maximum mobility, without ever causing pain. Coordination, agility, and strength are encouraged during exercises between patients and during lessons with the fencing master. The adapted fencing lesson ends with a cool-down period. Since different subjects experience different physical outcomes for the cancer and the subsequent treatment, the program must address these individual needs while respecting safety principles. Therefore, rest and water breaks will be provided when necessary, and simplified or less strenuous movements will be suggested as alternatives for those who find parts of the workout too challenging or unsafe. In addition, participants are encouraged to use both saber and foil, also depending on which master is holding the training session. In any case, the weapons used will be adapted in material to avoid excessive strain or fatigue as stated above. Moreover, the courses and masters allow 12 participants per session with a maximum ratio of 1 master per 2.5 participants. During the study protocol, participants are also encouraged to follow healthy diet and adhere to the standards of the Mediterranean diet through the prescription of a personalized diet by a nutritional biologist involved in the project. Conventional bioelectrical impedance (BIA) testing to assess body composition is also planned during protocol follow-ups. To assess adherence to the Mediterranean diet, the Prevención con Dieta Mediterránea (PREDIMED) questionnaire will be completed both at the beginning and at the end of the study (49). The participants of the FENICE study will be followed up by several specialists before and during the APA period (**Figure 3**). This study was approved by the ethic board of the Policlinico Tor Vergata of Univeristy of Rome and the enrollment will start in September 2024 and the recruitment will take an 18 months’ time window for the inclusion of the expected 150 participants.

### Inclusion and exclusion criteria

The inclusion and exclusion criteria for the FENICE trial are listed in **Table 3**.

**Table 3:** Inclusion and exclusion criteria.

Inclusion criteria	Exclusion criteria
The subject, at the time of diagnosis, must be between 18 and 70 years old	Subjects with recurrence of BC and treated with surgery, chemotherapy, radiotherapy, and hormone therapy
Subject affected by non-metastatic BC and treated with surgery for primary tumor	Life expectancy estimated to be less than three months by treating clinical team
Subject is able and willing to take part and be followed-up for at least 24 weeks	Unable and unwilling to consent to the study
Subject can carry out Adapted Physical Activity	Subject is, in the opinion of the Investigator, not suitable to participate in the study (e.g. surgical outcomes, severe co-morbidities, osteoporosis)
Subject has signed informed consent	Patient has existing impaired hepatic, renal or cardiorespiratory, Lymphatic function for which Adapted Physical Activity is contraindicated

### Recruitment and study population

Patients of any ethnicity who are at least 18 years old with diagnosis of non-metastatic BC and already treated with surgery for primary tumor are eligible. The recruiting physicians will check the in- and exclusion criteria and determine whether the patient is eligible for participation (see **Table 3**). Participation in the FENICE study is on a voluntary basis. Participants will be informed about the aims of the study both verbally and in writing and will have the opportunity to ask questions and refuse participation.

### Ethical considerations

The study will determine which group between subjects practicing APA and following Mediterranean diet (study arm) and those following the normal BC guidelines (standard of care) will benefit the most. Written informed consent, approved by an Italian Ethics Committee (R.S 100.23 5^th^ May 2023), will be obtained from each participant. Health complications for the participants will be addressed by the multidisciplinary team involved in the research and, if necessary, by contacting the patients’ MMG. If any complications that endanger the patient’s health, should arise the participant will be asked to withdraw from the study. Finally, subjects can leave the study at any time for any reason without any consequences, and, likewise, the research physicians can decide to withdraw a subject from the study for urgent medical reasons.

### Follow up visits

Participants will be included in the FENICE study for a total of 24 months and will be followed up differently based on the inclusion arm. In the study arm (APA and Mediterranean diet arm) patients will be monthly followed up for comorbidities, co-medication and herbal remedies, adverse events, and body mass index (BMI). Moreover, every three months also clinical evaluation, blood parameters, instrumental exams and QoL questionnaires will be carried out for the study arm patients. In the control arm, instead, all the mentioned parameters will be followed up every three months (see **Figure 2**).

### Data collection

Participants will be included in the study and will undergo several follow-up visits. During the follow-up period trained research nurses/data managers will contact patients at baseline (T=0) (± one week), and subsequently every four weeks (± two weeks), to go through a standardized questionnaire and to collect data on the outcomes of sequelae and clinical symptoms/AEs. In addition, all patients will be contacted by the research nurse/data manager at the end of the APA period (± two weeks from the end) to complete a last questionnaire. Research nurses/data managers additionally will collect required laboratory and or test results from participants’ (electronic) medical records. If a subject discontinues the APA and lifestyle interventions during the 12-week follow-up period, follow-up will continue.

The questions within the surveys will be performed to assess the occurrence and severity of any new symptoms experienced by the participants, and the global physical and psychological health after the beginning of APA. The questionnaires that will be administered to participants during the study period are listed in **Table 4**.

**Table 4:** Type of questionnaire that will be administered to breast cancer patients.

Objective	Questionnaire
**Disability**	The DASH (disabilities of the arm, shoulder, and hand measurement tool) questionnaire
**QoL**	EORTC qlq-C30 and EORTC qlq- BR23 questionnaire
**Fatigue**	The MFI 20 (Multidimensional fatigue inventory) questionnaire
**Anxiety-depression**	The HADS (Hospital anxiety and depression scale) questionnaire
**Physical activity and sedentary behavior**	The short version of the IPAQ (international physical activity questionnaire)
**Oedema assessment**	Disability scale for oedema

### Sample size

The hypothesis guiding the formal determination of the sample size proposes an expected improvement of at least 15 points in the mean DASH score. The DASH score is a valuable, pragmatic, and frequently utilized metric in individuals with various musculoskeletal disorders of the upper limb. Ranging from 0 to 100, it encompasses responses to 30 questions (https://orthotoolkit.com/dash/) An improvement of at least 15 points is considered clinically significant in enhancing functional ability. The statistical parameters are configured in a highly conservative manner, considering the non-pharmacological nature of the intervention (adapted physical activity) and the inherent inter-individual variability in response, particularly within the treated group (Group 1). Therefore, with alpha and beta errors set at 0.01, a standard deviation in Group 1 that is twice that of Group 2 (SD: 20 compared to 10), even exceeding the hypothesized difference (15 points), 56 patients per group are deemed necessary. Considering the enrollment capabilities of our group and potential physiological withdrawal of patients, the sample size has been increased to 150 total patients. The target population will be at least 18 years of age (of any ethnicity) with a diagnosis of non-metastatic breast cancer and already treated with surgery for the primary tumor. This total will be divided into two sub-groups by randomization in blocks of 75 patients each (intervention group, Group 1, and control group, Group 2). The first group will be distinguished by subjects treated with adapted physical activity rehabilitation and with correct lifestyle suggestions. The second group, on the other hand, will be characterized by standard care without physical activity rehabilitation or lifestyle suggestions.

### Statistical analysis

The descriptive statistical analyses, the central tendency, variability, symmetry and kurtosis will be calculated, all accompanied by appropriate graphical representations. Regarding the inferential analyses, we will continue with the verification of the parametric and non-parametric hypotheses, respectively for the quantitative variables with parametric tests such as: Student’s t-test, Fisher’s F-test, etc. and for categorical variables using non-parametric tests such as: chi-square, Mann Wittney’s U, Fisher’s exact test, etc. Only results with p-values ≤ 0.05 will be considered statistically significant. Principal component analysis (PCA) will be adopted to explore the relationships among secondary variables and identify patterns of improvement related to the APA intervention (50).

## Discussion

Regular physical activity performed during and after a BC treatment has been shown to improve quality of life (QoL) (34, 35). However, the type of exercise and the time spent on it are still based on patient’s will. Recently, a rehabilitation program was launched in France for women treated for BC that used adapted fencing as a rehabilitative activity. The RIPOSTE program aimed to offer adapted fencing to patients with BC (30, 43). In Italy, several authorities, including scientific associations (LILT – Lega Italiana per la Lotta ai Tumori), sports federations and Institutions (FIS – Federazione Italiana Scherma, CONI – Comitato Olimpico Nazionale Italiano) are committed to raising awareness of cancer patients and physicians on APA, diet, and correct lifestyle, throughout the promotion of observational studies, sporting, and popular events (“Nastro rosa”, “rema Roma per la vita”). With the FENICE study protocol, the initiative is intended to introduce the discipline of fencing into the rehabilitation program for women who have undergone surgery for breast cancer. Usually, after surgery, there is excessive internal rotation of the shoulder, which affects the patient’s posture, and fencing, especially the guard position, promotes an improvement of this condition. In addition, the range of motion resulting from increasingly higher parries under the guidance of the fencing master promotes the mobility of the arm and shoulder, which are limited by surgical scars and adhesions. The laterality of the exercise and the type of weapons used (foil and saber) allow constant work on mobilizing the operated shoulder and arm, especially since it is an upper-body activity that focuses on the upper body, from the belt to the head. (30, 43). Finally, the strong concentration and determination that fencing requires will certainly have a positive effect on image, mood, and mental strength, which are very often compromised following breast surgery. We firmly believe that the FENICE study by introducing adapted fencing in the near postoperative period, can be an effective tool to improve the physical and mental health of patients who have undergone surgery for BC. To date, the importance of adapted physical activity as a rehabilitation measure is emphasized in the literature, especially for cancer patients. In Italy, however, APA remains limited to local initiatives without being promoted by the health authorities. In some countries, such as France and the United Kingdom, APA is recognized as a prescription treatment for chronic diseases. As APA is therefore reimbursed by the health system, the sports doctor can prescribe a physical rehabilitation activity such as a medication or an examination. We hope that the FENICE protocol will be a pilot study for large-scale clinical trials that can lay the foundations for nationwide recognition of APA in the Italian healthcare system. Alongside the key role of the sports physician in APA implementation, this initiative might shed light on the role of physical activity and rehabilitation in improving WA of breast cancer patients since data from the literature are to date inconsistent (51). Currently, there is a lack of uniform and shared guidelines that can evaluate the Work Ability Index (WAI) through interventions or rehabilitative activities that support the patient’s return to work or, possibly, retention. Recently, the “Beyond Cancer” program by Sheppard and colleagues tried to overcome these barriers to sustainable return to work for women with breast cancer (52). In that perspective the FENICE study, as a tailored occupational rehabilitation intervention, may also include in its multidisciplinary network occupational physicians to assess the actual benefit of PA in promoting patients’ return to work and employability through dedicated questionnaires for participants in the adapted fencing group.

## Conclusion

In summary, the FENICE study protocol aims to introduce adapted Fencing and Mediterranean diet habits as suitable methods of postoperative BC rehabilitation. This innovative APA will be performed on 75 BC participants who will be closely followed by a multidisciplinary team of oncologists, physiatrists, nutritional biologists, radiotherapists, surgeons, cardiologists, gynecologists, psychologists, and Olympic fencing masters. We hypothesize successful adapted Fencing could improve BC patients’ physical status by reducing upper limb lymphedema and promoting a wider mobility of the operated limb. In addition to the physical improvement, the dietary recommendations (Mediterranean diet) together with workouts will positively affect patients’ lipid profile and the ratio of fat-free mass to fat mass. This study fits fully into the context of integrative and complementary medicine, which is increasingly finding a place in the care of the cancer patient. Together with the use of the most common biological CAMs (e.g., herbal products or botanicals, vitamins, minerals, probiotics, homeopathic products, and Chinese herbal remedies), also APA and proper nutrition (Mediterranean diet) fall into the large category of non-biological CAMs (53–56). The FENICE study protocol will be a further confirmation of an integrated approach to cancer treatment that requires multidisciplinary work and in-depth knowledge of this broad area.

## Ethics statement

The studies involving humans were approved byItalian Ethics Committee (R.S 100.23 5th May 2023); ethic board of the Policlinico Tor Vergata of Univeristy of Rome. The studies were conducted in accordance with the local legislation and institutional requirements. The participants provided their written informed consent to participate in this study.

## Author contributions

MB: Conceptualization, Visualization, Writing – original draft, Writing – review & editing, Methodology. DG: Conceptualization, Visualization, Writing – original draft, Writing – review & editing, Methodology. CF: Conceptualization, Visualization, Writing – review & editing. MR: Conceptualization, Visualization, Writing – review & editing. MM: Visualization, Writing – review & editing. GVi: Visualization, Writing – review & editing. FI: Visualization, Writing – review & editing. MS: Visualization, Writing – review & editing. GD: Visualization, Writing – review & editing. GS: Methodology, Visualization, Writing – original draft, Writing – review & editing. AO: Methodology, Visualization, Writing – review & editing. AN: Visualization, Writing – review & editing. MP: Visualization, Writing – review & editing. AB: Methodology, Visualization, Writing – original draft, Writing – review & editing. GVa: Methodology, Visualization, Writing – original draft, Writing – review & editing. OB: Conceptualization, Visualization, Writing – original draft, Writing – review & editing.

## References

[1] Cappellani A Di Vita M Zanghi A Cavallaro A Piccolo G Veroux M . Diet, obesity and breast cancer: an update. Front Biosci (Schol Ed). (2012) 4:90–108. doi: 10.2741/s253 22202045

[2] Caputo R Cianniello D Giordano A Piezzo M Riemma M Trovò M . Gene expression assay in the management of early breast cancer. Curr Medicinal Chem. (n.) 27:2826–39. doi: 10.2174/0929867326666191205163329 31804159

[3] Goodarzi E Beiranvand R Naemi H Pordanjani SR Khazaei Z . Geographical distribution Incidence and Mortality of Breast cancer and its relationship with the Human Development Index (HDI) - an ecology study in 2018. WCRJ. (2020) 7:e1468. doi: 10.32113/wcrj_20201_1468

[4] Mohamed RF Melek MI Eid S Morsy A . The correlation between increasing Body Mass Index and the incidence of local recurrence and distant metastasis in breast cancer patients. World Cancer Res J. (2023) 10:e2553. doi: 10.32113/wcrj_20235_2553

[5] Cancer screening program EU Recommendation . (2003). Available online at: https://eur-lex.europa.eu/legal-content/IT/TXT/?uri=OJ%3AL%3A2003%3A327%3ATOC (Accessed 6.16.24).

[6] Cancer (IARC) . Global cancer observatory(2022). Available online at: https://gco.iarc.fr/ (Accessed 6.18.24).

[7] I numeri del cancro in Italia . Associazione Italiana Registri Tumori(2023). Available online at: https://www.registri-tumori.it/cms/pubblicazioni/i-numeri-del-cancro-italia-2023 (Accessed 6.18.24).

[8] Tralongo P Pescarenico MG Surbone A Bordonaro S Berretta M DI Mari A . Physical needs of long-term cancer patients. Anticancer Res. (2017) 37:4733–46. doi: 10.21873/anticanres.11880 28870892

[9] Kaya T Karatepe AG Günaydn R Yetiş H Uslu A . Disability and health-related quality of life after breast cancer surgery: relation to impairments. South Med J. (2010) 103:37–41. doi: 10.1097/SMJ.0b013e3181c38c41 19996840

[10] Malicka I Barczyk K Hanuszkiewicz J Skolimowska B Woźniewski M . Body posture of women after breast cancer treatment. Ortop Traumatol Rehabil. (2010) 12:353–61. 20876929

[11] Koehler LA Blaes AH Haddad TC Hunter DW Hirsch AT Ludewig PM . Movement, function, pain, and postoperative edema in axillary web syndrome. Phys Ther. (2015) 95:1345–53. doi: 10.2522/ptj.20140377 PMC4595809 25977305

[12] Rostkowska E Bak M Samborski W . Body posture in women after mastectomy and its changes as a result of rehabilitation. Adv Med Sci. (2006) 51:287–97. 17357328

[13] Giacalone A Quitadamo D Zanet E Berretta M Spina M Tirelli U . Cancer-related fatigue in the elderly. Support Care Cancer. (2013) 21:2899–911. doi: 10.1007/s00520-013-1897-1 23852408

[14] Muraro E Furlan C Avanzo M Martorelli D Comaro E Rizzo A . Local high-dose radiotherapy induces systemic immunomodulating effects of potential therapeutic relevance in oligometastatic breast cancer. Front Immunol. (2017) 8:1476. doi: 10.3389/fimmu.2017.01476 29163540 PMC5681493

[15] Vinante L Avanzo M Furlan C Fiorica F Perin T Militello L . Ten daily fractions for partial breast irradiation. Long-term results of a prospective phase II trial. Breast J. (2019) 25:243–9. doi: 10.1111/tbj.2019.25.issue-2 30714257

[16] Hama Y Tate E . Survey on embarrassment of breast cancer patients receiving radiation therapy. World Cancer Res J. (2020) 7:e1607. doi: 10.32113/wcrj_20207_1607

[17] Muzzatti B Cattaruzza N Piccinin M Flaiban C Agostinelli G Berretta M . Cognitive function in long-term lymphoma survivors: relationship between subjective reports and objective assessments and with quality of life. Psychol Health Med. (2021) 26:968–79. doi: 10.1080/13548506.2020.1770815 32459120

[18] Yehya A Alshari O Alameri O Albals D Al-Taani G . Pre-pharmacological management: bi-screening for depression among breast cancer patients. World Cancer Res J. (2022) 9:e2233. doi: 10.32113/wcrj_20223_2233

[19] Camejo N Castillo C Santana D Argenzio L Amarillo D Herrera G . Arthralgia and myalgia associated with aromatase inhibitors: frequency and characterization in real-life patients. Ecancermedicalscience. (2024) 18:1697. doi: 10.3332/ecancer.2024.1697 38774562 PMC11108049

[20] Cerulli C Moretti E Grazioli E Emerenziani GP Murri A Tranchita E . Protective role of exercise on breast cancer-related osteoporosis in women undergoing aromatase inhibitors: a narrative review. Bone Rep. (2024) 21:101756. doi: 10.1016/j.bonr.2024.101756 38577250 PMC10990716

[21] Browall M Ahlberg K Karlsson P Danielson E Persson L-O Gaston-Johansson F . Health-related quality of life during adjuvant treatment for breast cancer among postmenopausal women. Eur J Oncol Nurs. (2008) 12:180–9. doi: 10.1016/j.ejon.2008.01.005 18343197

[22] Mokhtari-Hessari P Montazeri A . Health-related quality of life in breast cancer patients: review of reviews from 2008 to 2018. Health Qual Life Outcomes. (2020) 18:338. doi: 10.1186/s12955-020-01591-x 33046106 PMC7552560

[23] Wang L Hong BY Kennedy SA Chang Y Hong CJ Craigie S . Predictors of unemployment after breast cancer surgery: a systematic review and meta-analysis of observational studies. J Clin Oncol. (2018) 36:1868–79. doi: 10.1200/JCO.2017.77.3663 PMC6804906 29757686

[24] Gregorowitsch ML Bongard HJGD van den AM,C Young-Afat DA Haaring C Dalen TV . Self-reported work ability in breast cancer survivors; a prospective cohort study in the Netherlands. Breast. (2019) 48:45–53. doi: 10.1016/j.breast.2019.08.004 31493582

[25] Mishra SI Scherer RW Geigle PM Berlanstein DR Topaloglu O Gotay CC . Exercise interventions on health-related quality of life for cancer survivors. Cochrane Database Syst Rev. (2012) 2012:CD007566. doi: 10.1002/14651858.CD007566.pub2 22895961 PMC7387117

[26] Manneville F Rotonda C Conroy T Bonnetain F Guillemin F Omorou AY . The impact of physical activity on fatigue and quality of life during and after adjuvant treatment for breast cancer. Cancer. (2018) 124:797–806. doi: 10.1002/cncr.31108 29116645

[27] Campbell KL Winters-Stone KM Wiskemann J May AM Schwartz AL Courneya KS . Exercise guidelines for cancer survivors: consensus statement from international multidisciplinary roundtable. Med Sci Sports Exerc. (2019) 51:2375–90. doi: 10.1249/MSS.0000000000002116 PMC8576825 31626055

[28] McKenzie DC Kalda AL . Effect of upper extremity exercise on secondary lymphedema in breast cancer patients: a pilot study. J Clin Oncol. (2003) 21:463–6. doi: 10.1200/JCO.2003.04.069 12560436

[29] Giganti MG Tresoldi I Sorge R Melchiorri G Triossi T Masuelli L . Physical exercise modulates the level of serum MMP-2 and MMP-9 in patients with breast cancer. Oncol Lett. (2016) 12:2119–26. doi: 10.3892/ol.2016.4887 PMC4998658 27602150

[30] Omorou AY Peiffert D Rotonda C Van Hoye A Allado E Hily O . Adapted fencing for patients with invasive breast cancer: the RIPOSTE pilot randomized controlled trial. Front Sports Act Living. (2022) 4:786852. doi: 10.3389/fspor.2022.786852 35425895 PMC9002110

[31] Bloomquist K Adamsen L Hayes SC Lillelund C Andersen C Christensen KB . Heavy-load resistance exercise during chemotherapy in physically inactive breast cancer survivors at risk for lymphedema: a randomized trial. Acta Oncol. (2019) 58:1667–75. doi: 10.1080/0284186X.2019.1643916 31354000

[32] Gavala-González J Gálvez-Fernández I Mercadé-Melé P Fernández-García JC . Rowing training in breast cancer survivors: a longitudinal study of physical fitness. Int J Environ Res Public Health. (2020) 17:4938. doi: 10.3390/ijerph17144938 32659900 PMC7400517

[33] Johansson K Hayes S Speck RM Schmitz KH . Water-based exercise for patients with chronic arm lymphedema: a randomized controlled pilot trial. Am J Phys Med Rehabil. (2013) 92:312–9. doi: 10.1097/PHM.0b013e318278b0e8 23370582

[34] Patsou ED Alexias GD Anagnostopoulos FG Karamouzis MV . Effects of physical activity on depressive symptoms during breast cancer survivorship: a meta-analysis of randomised control trials. ESMO Open. (2017) 2:e000271. doi: 10.1136/esmoopen-2017-000271 29259819 PMC5729305

[35] Berretta M Facchini BA Garozzo D Necci V Taibi R Torrisi C . Adapted physical activity for breast cancer patients: shared considerations with two Olympic and world Italian sports champions. Eur Rev Med Pharmacol Sci. (2022) 26:5393–8. doi: 10.26355/eurrev_202208_29406 35993633

[36] Foti C Scordari M Vita G Imeshtari A Vanni G Torrisi C . Breast cancer rehabilitation and reconditioning. World Cancer Res J. (2023) 10:e2233. doi: 10.32113/wcrj_20239_2684

[37] Ibrahim EM Al-Homaidh A . Physical activity and survival after breast cancer diagnosis: meta-analysis of published studies. Med Oncol. (2011) 28:753–65. doi: 10.1007/s12032-010-9536-x 20411366

[38] Spei M-E Samoli E Bravi F La Vecchia C Bamia C Benetou V . Physical activity in breast cancer survivors: a systematic review and meta-analysis on overall and breast cancer survival. Breast. (2019) 44:144–52. doi: 10.1016/j.breast.2019.02.001 30780085

[39] Schmidt ME Chang-Claude J Vrieling A Seibold P Heinz J Obi N . Association of pre-diagnosis physical activity with recurrence and mortality among women with breast cancer. Int J Cancer. (2013) 133:1431–40. doi: 10.1002/ijc.v133.6 23444048

[40] Shachar SS Heiling H Muss HB Meghan D Wagoner CW Deal AM . Physical activity intervention in patients with metastatic breast cancer during active treatment: quality of life and function. Oncologist. (2022) 28:84–e70. doi: 10.1093/oncolo/oyac232 PMC9847548 36398879

[41] Jia T Liu Y Fan Y Wang L Jiang E . Association of healthy diet and physical activity with breast cancer: lifestyle interventions and oncology education. Front Public Health. (2022) 10:797794. doi: 10.3389/fpubh.2022.797794 35400043 PMC8984028

[42] Lahart IM Metsios GS Nevill AM Carmichael AR . Physical activity, risk of death and recurrence in breast cancer survivors: a systematic review and meta-analysis of epidemiological studies. Acta Oncol. (2015) 54:635–54. doi: 10.3109/0284186X.2014.998275 25752971

[43] Hasnaoui S Van Hoye A Soudant M Rotonda C Carvalho de Freitas A Peiffert D . Evaluating the feasibility and acceptability of an adapted fencing intervention in breast cancer surgery post-operative care: the RIPOSTE pilot randomized trial. Front Oncol. (2024) 14:1335442. doi: 10.3389/fonc.2024.1335442 38665959 PMC11043494

[44] Woltmann ML Foster C Porcari JP Camic CL Dodge C Haible S . Evidence that the talk test can be used to regulate exercise intensity. J Strength Cond Res. (2015) 29:1248–54. doi: 10.1519/JSC.0000000000000811 25536539

[45] Hagströmer M Oja P Sjöström M . The International Physical Activity Questionnaire (IPAQ): a study of concurrent and construct validity. Public Health Nutr. (2006) 9:755–62. doi: 10.1079/PHN2005898 16925881

[46] Lear SA Brozic A Myers JN Ignaszewski A . Exercise stress testing. Sports Med. (1999) 27:285–312. doi: 10.2165/00007256-199927050-00002 10368877

[47] Haskell WL Lee I-M Pate RR Powell KE Blair SN Franklin BA . Physical activity and public health: updated recommendation for adults from the American College of Sports Medicine and the American Heart Association. Med Sci Sports Exerc. (2007) 39:1423–34. doi: 10.1249/mss.0b013e3180616b27 17762377

[48] Garber CE Blissmer B Deschenes MR Franklin BA Lamonte MJ Lee I-M . American College of Sports Medicine position stand. Quantity and quality of exercise for developing and maintaining cardiorespiratory, musculoskeletal, and neuromotor fitness in apparently healthy adults: guidance for prescribing exercise. Med Sci Sports Exerc. (2011) 43:1334–59. doi: 10.1249/MSS.0b013e318213fefb 21694556

[49] Martínez-González MA García-Arellano A Toledo E Salas-Salvadó J Buil-Cosiales P Corella D . A 14-item mediterranean diet assessment tool and obesity indexes among high-risk subjects: the PREDIMED trial. PloS One. (2012) 7:e43134. doi: 10.1371/journal.pone.0043134 22905215 PMC3419206

[50] Ringnér M . What is principal component analysis? Nat Biotechnol. (2008) 26:303–4. doi: 10.1038/nbt0308-303 18327243

[51] Algeo N Bennett K Connolly D . Rehabilitation interventions to support return to work for women with breast cancer: a systematic review and meta-analysis. BMC Cancer. (2021) 21:895. doi: 10.1186/s12885-021-08613-x 34353286 PMC8340442

[52] Sheppard DM O’Connor M Jefford M Lamb G Frost D Ellis N . Beyond cancer” Rehabilitation program to support breast cancer survivors to return to health, wellness and work: feasibility study outcomes. Curr Oncol. (2023) 30:2249–70. doi: 10.3390/curroncol30020174 PMC9956005 36826135

[53] Abdelbasset WK Ibrahim AA Alsubaie SF Alrawaili SM Althomali OW Hussein HM . Awareness and knowledge of breast cancer rehabilitation among Saudi Arabia physical therapists. Eur Rev Med Pharmacol Sci. (2023) 27:5370–7. doi: 10.26355/eurrev_202306_32771 37401271

[54] Berretta M Montopoli M Cazzavillan S Ceppa F Santagà D Berti C . Integrative multidisciplinary approaches by the Integrative Medicine Research Group (IMRG): a new frontier in cancer setting. Eur Rev Med Pharmacol Sci. (2023) 27:10507–21. doi: 10.26355/eurrev_202311_34327 37975374

[55] Berretta M Morra A Taibi R Monari F Maurea N Ippolito M . Improved survival and quality of life through an integrative, multidisciplinary oncological approach: pathophysiological analysis of four clinical cancer cases and review of the literature. Front Pharmacol. (2022) 13:867907. doi: 10.3389/fphar.2022.867907 35784762 PMC9243589

[56] Berretta M Rinaldi L Taibi R Tralongo P Fulvi A Montesarchio V . Physician attitudes and perceptions of complementary and alternative medicine (CAM): a multicentre Italian study. Front Oncol. (2020) 10:594. doi: 10.3389/fonc.2020.00594 32411599 PMC7202223

